# 

**Published:** 1852-01-01

**Authors:** 


### THE JOURNAL

OF

### PSYCHOLOGICAL MEDICINE

AND

### MENTAL PATHOLOGY.

EDITED BY

FORBES WINSLOW, M.D.

VOL. V.

Surgeon Genl's Office
LIBRARY.
22049
Washington, D.C.

LONDON:
JOHN CHURCHILL, PRINCES STREET, SOHO.
MDCCCLII.

A Case for Medical Charity.—A general practitioner, resident in the country, has requested us to make known his case to the profession. The party referred to is at this moment in great pecuniary difficulties. An attack of paralysis, from which he has for some period suffered, has completely crippled all his efforts to pursue his profession. He writes that he is almost in want of the necessaries of life, and that the work-house stares him in the face. The Editor of this journal has, by personal observation, ascertained the truth of the gentleman's assertions, and he can conscientiously recommend the case to the kind hearts of his readers. It will afford him much pleasure to place any of his friends in communication with the party.

